# Diagnosis of acute myocardial infarction using a combination of circulating circular RNA cZNF292 and clinical information based on machine learning

**DOI:** 10.1002/mco2.299

**Published:** 2023-06-13

**Authors:** Qiulian Zhou, Jes‐Niels Boeckel, Jianhua Yao, Juan Zhao, Yuzheng Bai, Yicheng Lv, Meiyu Hu, Danni Meng, Yuan Xie, Pujiao Yu, Peng Xi, Jiahong Xu, Yi Zhang, Stefanie Dimmeler, Junjie Xiao

**Affiliations:** ^1^ Institute of Geriatrics (Shanghai University) Affiliated Nantong Hospital of Shanghai University (The Sixth People's Hospital of Nantong) and School of Life Science Shanghai University Nantong China; ^2^ Cardiac Regeneration and Ageing Lab Institute of Cardiovascular Sciences Shanghai Engineering Research Center of Organ Repair, School of Medicine Shanghai University Shanghai China; ^3^ Institute for Cardiovascular Regeneration University Frankfurt Frankfurt Germany; ^4^ Klinik und Poliklinik für Kardiologie Universitätsklinikum Leipzig Leipzig Germany; ^5^ Department of Cardiology Shanghai Tenth People's Hospital Tongji University School of Medicine Shanghai China; ^6^ Department of Cardiology Shigatse People's Hospital Tibet China; ^7^ School of Pharmacy Shanghai University of Traditional Chinese Medicine Shanghai China; ^8^ Department of Cardiology Tongji Hospital Tongji University School of Medicine Shanghai China

**Keywords:** acute myocardial infarction, biomarker, circular RNA, cZNF292, machine learning

## Abstract

Circulating circular RNAs (circRNAs) are emerging as novel biomarkers for cardiovascular diseases (CVDs). Machine learning can provide optimal predictions on the diagnosis of diseases. Here we performed a proof‐of‐concept study to determine if combining circRNAs with an artificial intelligence approach works in diagnosing CVD. We used acute myocardial infarction (AMI) as a model setup to prove the claim. We determined the expression level of five hypoxia‐induced circRNAs, including cZNF292, cAFF1, cDENND4C, cTHSD1, and cSRSF4, in the whole blood of coronary angiography positive AMI and negative non‐AMI patients. Based on feature selection by using lasso with 10‐fold cross validation, prediction model by logistic regression, and ROC curve analysis, we found that cZNF292 combined with clinical information (CM), including age, gender, body mass index, heart rate, and diastolic blood pressure, can predict AMI effectively. In a validation cohort, CM + cZNF292 can separate AMI and non‐AMI patients, unstable angina and AMI patients, acute coronary syndromes (ACS), and non‐ACS patients. RNA stability study demonstrated that cZNF292 was stable. Knockdown of cZNF292 in endothelial cells or cardiomyocytes showed anti‐apoptosis effects in oxygen glucose deprivation/reoxygenation. Thus, we identify circulating cZNF292 as a potential biomarker for AMI and construct a prediction model “CM + cZNF292.”

## Graphical abstract

Artificial intelligence provides a distinct view on the diagnosis of diseases. Our data prove a potential opportunity to improve clinical assessment and guide clinical decision‐making by using a model based on easily available clinical data and circulating circRNAs in acute myocardial infarction. Thus, combining circRNAs biomarkers with an artificial intelligence approach could be used to diagnose cardiovascular disease.

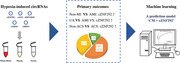

## 1 INTRODUCTION

Cardiovascular disease (CVD) is a leading threat to human lives, causing great morbidity and mortality worldwide.^1^, ^2^ Accurate and early diagnoses combined with appropriate therapeutic strategy would significantly reduce mortality and improve prognosis.^3^, ^4^ Clinical diagnosis of acute myocardial infarction (AMI) is based on electrocardiography combined with cardiac troponins, myoglobin, and creatine kinase‐MB (CK‐MB) isoenzymes.^5^ However, the investigation of novel biomarkers for the diagnosis of AMI continues.

Circulating noncoding RNAs have emerged as a promising biomarker for CVD.^6^ Among them, circular RNAs (CircRNAs) represent a compelling component.^7^, ^8^ CircRNAs are endogenous, abundant noncoding RNAs mainly formed by back‐splice events, and they exist in the form of closed continuous loops.^9^ CircRNAs are present in a tissue and developmental stage‐specific manner.^9^, ^10^ CircRNAs regulate many essential biological processes, including proliferation, apoptosis, autophagy, and metabolism.^11^, ^12^ Dysregulated circRNAs contribute to several human diseases, including CVDs.^9^, ^13^ Interestingly, circRNAs are present in a stable form in the circulation, and its change may reflect disease stage.^10^, ^14^ Thus, circulating circRNAs are emerging as novel biomarkers for human diseases.^14^

Several circulating circRNAs have been related to CVDs. CircRNA_0124644 and circRNA_0098964 have been found to be a potential diagnostic biomarker of coronary artery disease (CAD).^15^ Circulating circRNAs DNAJC6, TMEM56, and MBOAT2 were suggested as biomarkers for hypertrophic obstructive cardiomyopathy,^16^ whereas circulating circRNA_004183, circRNA_079265, and circRNA_105039 might be used for the diagnosis of congenital heart disease in children.^17^ Moreover, for acute ischemia stroke, the circulating circRNAs circFUNDC1, circPDS5B, and circCDC14A might be suitable biomarker candidates.^18^ Besides, blood MICRA level was found to be lower in AMI patients and can be used for risk stratification.^19^, ^20^ Five circRNAs, including cZNF292, cAFF1, cDENND4C, cTHSD1, and cSRSF4, have been characterized as hypoxia‐induced circRNAs in endothelial cells.^21^ However, if these five circRNAs in circulation could serve as a valuable biomarker for AMI is undetermined. In addition, the role of cZNF292 in cardiac diseases has not been investigated.

Recently, the development of machine learning and artificial intelligence provides a distinct view on the diagnosis and treatment of complex diseases, enables scientists to identify new patterns and information from datasets, and also helps clinicians diagnose complex diseases more effectively and efficiently.^22^ Machine learning can provide optimal predictions on the diagnosis of diseases.^23^ Lasso is widely used for feature selection and logistic regression for classification and disease prediction.^24^, ^25^ Here we performed a proof‐of‐concept study to determine if combining circRNAs with an artificial intelligence approach works in diagnosing CVD.

AMI was used as a model setup to proof our claim. In this study, we identified circulating circRNA cZNF292 as a potential biomarker for AMI, and we constructed a prediction model “CM (clinical information) + cZNF292” by integrating cZNF292 and CM based on lasso and logistic regression. We compared “CM + cZNF292” to other models by ROC curve analysis, and our model always had the highest area under curve (AUC) value. Besides, the “CM + cZNF292” model only needs the information of basic CM, including age, gender, body mass index (BMI), heart rate, and diastolic blood pressure (DBP), which can be easily accessed without asking the patients orally.

## 2 RESULTS

### 2.1 Expression levels of circulating hypoxia‐induced circRNAs in the discovery cohort

To analyze the potential function as biomarkers, we first determined the changes of the hypoxia‐induced circRNAs, including cZNF292, cAFF1, cDENND4C, cTHSD1, and cSRSF4, in the circulation AMI and non‐AMI patients.^21^

**FIGURE 1:**
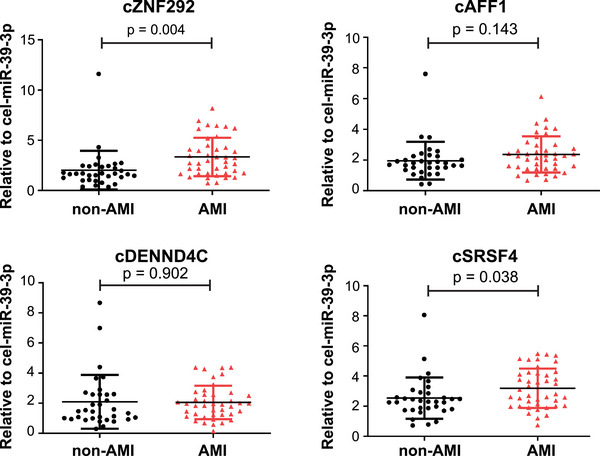
Quantitative reverse‐transcription polymerase chain reaction (qRT‐PCR) analysis of whole blood levels of cZNF292, cAFF1, cDENND4C, and cSRSF4 in acute myocardial infarction (AMI) and non‐AMI patients. *n* = 33 for non‐AMI (here refers to negative for coronary angiography); *n* = 42 for AMI (here refers to positive for coronary angiography).

The discovery cohort included a total of 75 patients. Based on the results of coronary angiography, 42 were positive (diagnosed as AMI), whereas 33 were negative (diagnosed as non‐AMI). AMI patients have higher DBP, neutrophil (%), N‐terminal pro‐B‐type natriuretic peptide (NT‐proBNP), high sensitivity cardiac troponin T, myoglobin, CK‐MB, C‐reactive protein, total bilirubin (TB), aspartate aminotransferase (AST), CK, and creatinine as compared to those not (Table 1). The levels of five hypoxia‐induced circRNAs, including cZNF292, cAFF1, cDENND4C, cTHSD1, and cSRSF4, were determined in whole blood, serum, and plasma. All analyzed hypoxia‐induced circRNAs, except cTSHSD1, were detected in whole blood specimens. Levels of cAFF1 and cSRSF4 were detected in serum and plasma, whereas cZNF292 and cDENND4C were not (Figures S1–S3). Therefore, we chose whole blood to detect the four hypoxia‐induced circRNAs, including cZNF292, cAFF1, cDENND4C, and cSRSF4, in AMI and non‐AMI patients as determined by coronary angiography. Compared to the non‐AMI patients, we found higher levels of circulating cZNF292 and cSRSF4 in AMI patients, whereas levels of cAFF1 and cDENND4C were not changed (Figure 1).

**TABLE 1:** The characteristics of non‐acute myocardial infarction (AMI) and AMI patients in the discovery cohort.

Variable	non‐AMI	AMI	*p* Value
*N*	33	42	
**Demographic characteristics**			
Male sex (% male)	20 (60.6)	30 (71.4)	
Age (years)	60.85 ± 10.62	64.76 ± 10.61	0.117
BMI (kg/m^2^)	25.48 ± 3.60	24.58 ± 2.68	0.218
Heart rate (bpm)	80.67 ± 15.11	79.83 ± 24.17	0.863
SBP (mmHg)	137.67 ± 16.58	140.95 ± 23.10	0.476
DBP (mmHg)	76.85 ± 12.98	84.48 ± 16.46	0.032
**Biochemical examinations**			
Hemoglobin (g/dL)	143.42 ± 24.26	137.69 ± 19.57	0.268
RBC (×10^12/L)	4.53 ± 0.73	4.52 ± 0.63	0.931
WBC (×10^9/L)	3.55–5.96 (5.00)	8.05–11.48 (9.77)	0.150
BPC (×10^9/L)	239.03 ± 75.87	223.66 ± 74.59	0.390
Neutrophil (%)	51.86 ± 19.39	71.91 ± 17.53	<0.001
Potassium (mM)	3.60–4.10 (3.90)	3.50–4.10 (3.98)	0.379
Sodium (mM)	139.50–144.50 (142.00)	138.00–141.00 (140.00)	0.770
NT‐proBNP (pg/mL)	17.30–107.62 (43.82)	86.45–1731.00 (494.60)	0.010
hs‐cTnT (ng/mL)	0.005–0.011 (0.006)	0.065–2.275 (0.309)	<0.001
Myoglobin (ng/mL)	21.00–33.23 (23.69)	59.66–371.90 (149.10)	<0.001
CK‐MB (ng/mL)	1.08–2.22 (1.69)	2.59–45.33 (14.18)	<0.001
d‐dimer (mg/L)	0.23–0.48 (0.34)	0.22–0.72 (0.36)	0.103
CRP (mg/dL)	0.84–3.23 (1.55)	0.90–17.20 (3.23)	0.027
TB (μmol/L)	9.60 ± 3.46	14.65 ± 7.81	0.002
DB (μmol/L)	2.56–5.35 (3.70)	2.80–5.85 (4.50)	0.483
TC (mmol/L)	4.15 ± 1.03	4.20 ± 1.06	0.856
TG (mmol/L)	0.98–1.67 (1.34)	0.91–1.77 (1.37)	0.267
HDL‐C (mM)	0.85–1.55 (1.09)	0.83–1.13 (0.91)	0.061
LDL‐C (mM)	2.00–3.10 (2.20)	1.97–3.41 (2.72)	0.329
ALT (U/L)	15.60–45.00 (27.30)	25.15–52.00 (37.35)	0.187
AST (U/L)	17.60–29.98 (23.45)	34.73–135.03 (60.55)	<0.001
CK (ng/mL)	58.25–112.08 (82.10)	126.00–503.90 (224.60)	0.004
Creatinine (μM)	68.61 ± 15.82	81.71 ± 24.30	0.008
Uric acid (μM)	384.68 ± 107.57	343.62 ± 97.06	0.092

*Note*: All data were presented as an absolute number (percentage), the mean (standard deviation), or the 25th–75th percentile (median).

Abbreviations: ALT, alanine amino transferase; AST, aspartate amino transferase; BMI, body mass index; BPC, blood platelet cell; CK, creatine kinase; CRP, C‐reactive protein; DB, direct bilirubin; DBP, diastolic blood pressure; HDL‐C, high‐density lipoprotein; hs‐cTnT, high sensitivity cardiac troponin T; LDL‐C, low density lipoprotein; NT‐proBNP, N‐terminal pro‐B‐type natriuretic peptide; RBC, red blood cell; SBP, systolic blood pressure; TB, total bilirubin; TC, total cholesterol; TG, triglyceride; WBC, white blood cell.

These results suggest that circulating cZNF292 and cSRSF4 may be potential candidates as biomarkers for the diagnosis of AMI.

### 2.2 Constructing a prediction model using the training cohort

**FIGURE 2:**
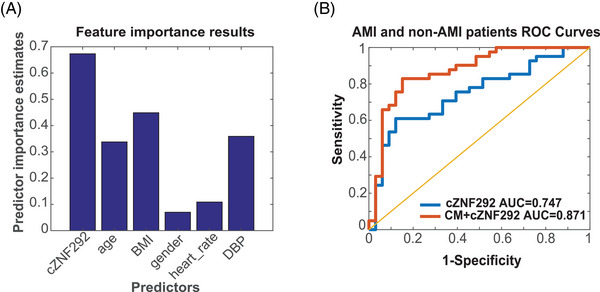
Feature importance test and ROC curve for discovery cohort: (A) the feature importance test results based on Bagging Decision Trees; (B) ROC curve for cZNF292 (blue line) and clinical information (CM) + cZNF292 (red line). CM means a clinical model, including age, gender, body mass index (BMI), heart rate, and diastolic blood pressure (DBP). *n* = 33 for non‐acute myocardial infarction (AMI) (here refers to negative for coronary angiography); *n* = 42 for AMI (here refers to positive for coronary angiography).

To test the potential novel biomarkers for AMI, we constructed a prediction model in the training cohort. We first performed feature selection by using lasso based on CM and cZNF292 and cSRSF4 levels, which were significantly higher in the whole blood of AMI patients. We used 10‐fold cross validation to identify the best estimator parameters (Figure S4). According to the result of lasso, six variables were selected as features based on the values of estimator parameters (Table S1), which were cZNF292, age, gender, BMI, heart rate, and DBP. We use CM + cZNF292 to indicate the six features, where CM includes age, gender, BMI, heart rate, and DBP. In contrast, the coefficients of circRNA cSRSF4 and the CM of systolic blood pressure (SBP) are 0, indicating that they are not predictors (Table S1). Therefore, these two variables are not included in our model. To further evaluate the features, we used Bagging Decision Trees to test the importance of each single feature. Consistently, we found that cZNF292 was the most important feature followed by BMI (Figure 2A). Then we used logistic regression to make predictions based on different features and feature combinations. The ROC curve analysis was used to analyze the diagnostic accuracy of circulating cZNF292 and cSRSF4 for AMI (Table S2 and Figure 2B). For circulating cZNF292, the AUC was 0.747 (95% confidence interval, 0.619–0.852; *p* < 0.01). For circulating cSRSF4, the AUC was 0.672 (95% confidence interval, 0.541–0.780; *p* < 0.01). For the CM, the AUC were all lower than cZNF292 (Table S2). However, when we used the CM + cZNF292 features to improve the prediction, the AUC was 0.871, which was obviously higher than the values by using each feature individually (Figure 2B and Table S2). For comparison, we also use cZNF292 combined with only one CM to do prediction, and the values of AUC were all lower than the results of CM + cZNF292 (Table S2).

These results suggest that the performance of a circulating noncoding RNA such as cZNF292 in the diagnosing of AMI can be improved by combining it with CM in an artificial intelligence prediction model.

### 2.3 Stability analyses of cZNF292

**FIGURE 3:**
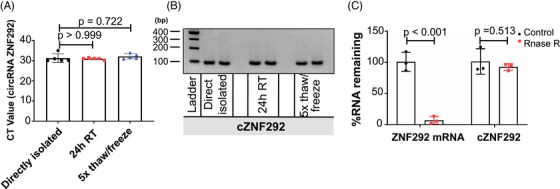
cZNF292 is stable in human whole blood: (A) the mean CT value of cZNF292 in human whole blood treated with directly isolated, incubated for 24 h at RT, or frozen and thawed for five cycles before isolating RNA. *n* = 5 for directly isolated; *n* = 5 for 24 h RT; *n* = 5 for 5× thaw/freeze; (B) semiquantitative polymerase chain reaction (PCR) analysis of cZNF292 in human whole blood; (C) quantification of cZNF292 and ZNF292 after RNase R digestion by qPCR. *n* = 3 for Control; *n* = 3 for RNase R.

To further support the use of cZNF292 as a biomarker, we performed stability analyses of the cZNF292 RNA molecule. Therefore, the stability of the cZNF292 molecule was determined in RNA samples isolated from five healthy individuals by either incubation at room temperature for 24 h or subjecting it to five cycles of freezing and thawing. These two treatments had no effect on the detectable level of cZNF292 (Figure 3A,B and Figure S5), demonstrating that cZNF292 is stable in human whole blood. Further RNA stability analyses revealed that cZNF292 is more resistant to RNase R treatment than linear ZNF292 mRNA (Figure 3C). These results suggest that cZNF292 is stable and can potentially be used as a biomarker.

### 2.4 Prediction in validation cohort

To confirm that the model CM + cZNF292 can be used to diagnose AMI and also prove the concept that combining circRNAs biomarkers with an artificial intelligence approach is promising in diagnosing CVD, we tested our findings in an additional cohort of patients with AMI. The validation cohort included a total of 138 patients: 75 MI, 15 unstable angina (UA), and 48 stable angina.

**FIGURE 4:**
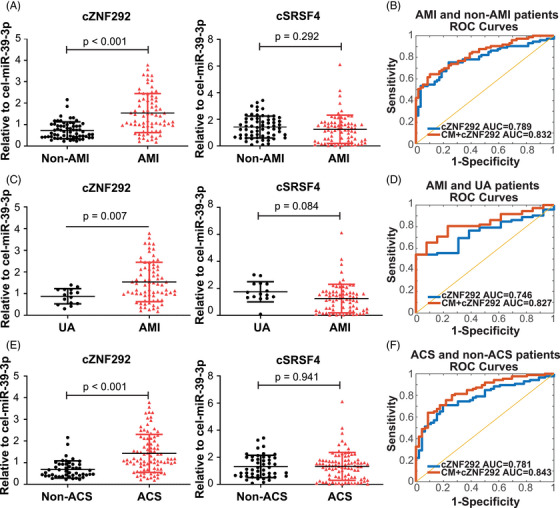
Quantitative reverse‐transcription polymerase chain reaction (qRT‐PCR) analysis and ROC curve of whole blood levels of cZNF292 in validation study: (A) qRT‐PCR analysis of whole blood levels of cZNF292 and cSRSF4 in non‐acute myocardial infarction (AMI) (here refers to unstable angina (UA) or stable angina) and AMI patients. *n* = 63 for non‐AMI; *n* = 75 for AMI; (B) ROC curve of AMI and non‐AMI patients for cZNF292 (blue line) and clinical information (CM) + cZNF292 (red line). *n* = 63 for non‐AMI; *n* = 75 for AMI; (C) qRT‐PCR analysis of whole blood levels of cZNF292 and cSRSF4 in UA and AMI patients. *n* = 15 for UA; *n* = 75 for AMI; (D) ROC curve of AMI and UA patients for cZNF292 (blue line) and CM + cZNF292 (red line). *n* = 15 for UA; *n* = 75 for AMI; (E) qRT‐PCR analysis of whole blood levels of cZNF292 and cSRSF4 in non‐acute coronary syndromes (ACS) (here refers to stable angina) and ACS patients (including UA and AMI). *n* = 48 for non‐ACS; *n* = 90 for ACS. (F) ROC curve of ACS and non‐ACS patients for cZNF292 (blue line) and CM + cZNF292 (red line). *n* = 48 for non‐ACS; *n* = 90 for ACS. CM means a clinical model, including age, gender, body mass index (BMI), heart rate, and diastolic blood pressure (DBP).

As compared to non‐AMI patients (here refers to UA or stable angina), AMI patients have lower sodium and BMI, higher red blood cell (RBC), leukocyte (WBC), blood platelet (BPC), neutrophil (%), NT‐proBNP, hs‐cTnI, myoglobin, CK‐MB, TB, total cholesterol, low‐density lipoprotein (LDL‐C), alanine aminotransferase, AST, and CK (Table 2). Compared to the non‐AMI patients, the levels of circulating cZNF292 were higher in AMI patients, whereas the levels of cSRSF4 were not changed (Figure 4A). The ROC curves for cZNF292 reflected strong separation between AMI and non‐AMI patients, with an AUC of 0.789 (95% confidence interval 0.702–0.861) (Figure 4B). The maximal value of the Youden index (*J*) was used as threshold to select the optimal cutoff point.^26^ With the maximal value of the Youden index as 0.516, the corresponding sensitivity and specificity of cZNF292 were 75.34% and 76.27%, respectively. Then we used features CM + cZNF292 to make prediction. The AUC was 0.832 (Figure 4B), which was higher compared to cZNF292 combined with only one CM (Table S3).

**TABLE 2:** The characteristics of acute myocardial infarction (AMI), non‐AMI, and unstable angina (UA) patients in validation cohort.

Variable	AMI	Non‐AMI	UA	*p* Value (non‐AMI vs. AMI)	*p* Value (UA vs. AMI)
*N*	75	63	15		
**Demographic characteristics**					
Male sex (% male)	63 (84.0)	43 (68.3)	13 (86.7)		
Age (years)	62.60 ± 11.64	64.29 ± 12.28	68.60 ± 10.92	0.411	0.069
BMI (kg/m^2^)	19.58–22.00 (20.60)	19.40–24.95 (22.35)	18.00–23.38 (19.50)	0.025	0.698
Heart rate (bpm)	71.00–90.00 (80.00)	74.00–85.00 (80.00)	80.00–86.00 (80.00)	0.668	0.427
SBP (mmHg)	130.12 ± 22.66	129.87 ± 17.21	131.80 ± 14.48	0.942	0.784
DBP (mmHg)	70.00–86.00 (78.00)	74.00–82.00 (80.00)	80.00–88.00 (80.00)	0.592	0.258
**Biochemical examinations**					
Hemoglobin (g/dL)	134.00–157.00 (148.00)	125.00–144.00 (133.00)	130.00–149.00 (140.00)	0.732	0.172
RBC (×10^12/L)	4.74 ± 0.51	4.41 ± 0.46	4.61 ± 0.49	<0.001	0.372
WBC (×10^9/L)	9.18 ± 2.58	6.36 ± 1.90	6.89 ± 1.36	<0.001	<0.001
BPC (×10^9/L)	220.19 ± 59.23	187.11 ± 53.14	201.20 ± 57.92	0.001	0.258
Neutrophil (%)	70.07 ± 13.15	62.07 ± 9.47	67.23 ± 8.28	<0.001	0.288
Potassium (mM)	3.81 ± 0.37	3.70 ± 0.41	3.73 ± 0.40	0.096	0.454
Sodium (mM)	140.25 ± 2.94	142.47 ± 2.04	141.83 ± 2.02	<0.001	0.050
NT‐proBNP (pg/mL)	499.60–2012.25 (1044.00)	38.80–276.05 (74.60)	48.90–1173.00 (200.90)	<0.001	0.112
hs‐cTnI (ng/mL)	13.184–77.838 (40.052)	0.010–0.031 (0.0130)	0.011–0.068 (0.033)	<0.001	<0.001
Myoglobin (ng/mL)	86.70–485.60 (154.00)	16.10–31.23 (21.70)	15.80–34.00 (27.70)	<0.001	<0.001
CK‐MB (ng/mL)	48.60–285.80 (161.10)	1.10–2.10 (1.40)	1.50–2.50 (1.90)	<0.001	<0.001
d‐dimer (mg/L)	0.19–0.52 (0.30)	0.19–0.46 (0.26)	0.21–0.87 (0.43)	0.605	0.749
CRP (mg/dL)	0.94–5.47 (2.72)	0.20–3.14 (1.49)	0.38–3.14 (1.64)	0.775	0.119
TB (μmol/L)	17.12 ± 7.60	13.71 ± 5.63	13.75 ± 3.18	0.004	0.007
DB (μmol/L)	1.00–3.80 (2.60)	1.80–3.50 (2.70)	2.20–3.80 (2.90)	0.662	0.810
TC (mmol/L)	4.78 ± 1.29	4.19 ± 1.10	4.00 ± 1.30	0.005	0.035
TG (mmol/L)	0.96–1.76 (1.28)	1.02–1.90 (1.22)	0.88–1.87 (1.22)	0.471	0.300
HDL‐C (mM)	1.03 ± 0.21	1.03 ± 0.24	0.98 ± 0.21	0.934	0.348
LDL‐C (mM)	3.34 ± 1.06	2.82 ± 0.86	2.69 ± 1.02	0.002	0.030
TP (g/L)	66.76 ± 6.22	66.41 ± 5.87	65.69 ± 7.89	0.740	0.564
ALB (g/L)	38.86 ± 3.40	39.73 ± 4.42	38.21 ± 6.34	0.195	0.705
ALT (U/L)	25.00–46.00 (35.00)	13.00–23.00 (17.00)	14.00–23.00 (16.00)	0.003	0.019
AST (U/L)	32.00–183.00 (83.00)	16.00–25.00 (19.00)	17.00–24.00 (19.00)	<0.001	<0.001
CK (ng/mL)	216.00–1358.00 (644.00)	54.00–107.00 (74.00)	48.00–109.00 (65.00)	<0.001	<0.001
BUN (mM)	4.28–6.40 (5.52)	4.49–6.52 (5.30)	3.97–6.70 (5.20)	0.798	0.580
Creatinine (μM)	68.00–92.00 (81.00)	67.00–87.00 (77.00)	62.00–92.00 (82.00)	0.122	0.676
Uric acid (μM)	372.96 ± 106.21	388.48 ± 128.74	432.27 ± 170.11	0.439	0.081

*Note*: All data were presented as an absolute number (percentage), the mean (standard deviation), or the 25th–75th percentile (median).

ALB, albumin; ALT, alanine amino transferase; AST, aspartate amino transferase; BMI, body mass index; BPC, blood platelet cell; BUN, blood urea nitrogen; CK, creatine kinase; CRP, C‐reactive protein; DB, direct bilirubin; DBP, diastolic blood pressure; HDL‐C, high‐density lipoprotein; LDL‐C, low density lipoprotein; NT‐proBNP, N‐terminal pro‐B‐type natriuretic peptide; RBC, red blood cell; SBP, systolic blood pressure; TB, total bilirubin; TC, total cholesterol; TG, triglyceride; TP, total protein; WBC, white blood cell.

Compared to the UA patients, circulating cZNF292 was significantly higher in AMI patients, whereas cSRSF4 was not changed (Figure 4C). The ROC curves of cZNF292 reflect a strong separation between AMI and UA patients, with an AUC of 0.746 (95% confidence interval 0.612–0.844) (Figure 4D). With the maximal value of the Youden index as 0.528, the corresponding sensitivity and specificity of cZNF292 were 52.78% and 100%, respectively. Then we used features CM + cZNF292 to make prediction. The AUC was 0.827 (Figure 4D), which was higher than the results when we used cZNF292 combined with only one CM (Table S4).

As compared to non‐acute coronary syndromes (ACS) patients, ACS patients have lower BMI, sodium, albumin (ALB), and higher RBC, WBC, BPC, neutrophil (%), NT‐proBNP, hs‐cTnI, myoglobin, CK‐MB, TB, LDL‐C, AST, and CK (Table 3). Compared to the non‐ACS patients, circulating cZNF292 was significantly higher in ACS patients, whereas cSRSF4 was not changed (Figure 4E). The ROC curves of cZNF292 reflected strong separation between ACS and non‐ACS patients, with an AUC of 0.781 (95% confidence interval 0.694–0.851) (Figure 4F). With the maximal value of the Youden index as 0.513, the corresponding sensitivity and specificity of cZNF292 were 70.93% and 80.43%, respectively. Then we used features CM + cZNF292 to make prediction. The AUC was 0.843 (Figure 4F), which was higher than the results when we used cZNF292 combined with only one CM (Table S5).

**TABLE 3:** The characteristics of non‐acute coronary syndromes (ACS) and ACS patients in validation cohort.

Variable	Non‐ACS	ACS	*p* Value
*N*	48	90	
**Demographic characteristics**			
Male sex (% male)	30 (62.5)	76 (84.4)	
Age (years)	62.92 ± 12.48	63.60 ± 11.68	0.751
BMI (kg/m^2^)	20.07–25.38 (23.48)	19.50–22.25 (20.60)	0.003
Heart rate (bpm)	70.25–84.25 (79.50)	72.00–90.00 (80.00)	0.247
SBP (mmHg)	129.27 ± 18.08	130.40 ± 21.45	0.757
DBP (mmHg)	74.00–80.75 (79.50)	71.00–86.00 (80.00)	0.852
**Biochemical examinations**			
Hemoglobin (g/dL)	122.25–143.75 (130.50)	133.00–156.00 (146.00)	0.671
RBC (×10^12/L)	4.34 ± 0.44	4.72 ± 0.51	<0.001
WBC (×10^9/L)	6.19 ± 2.02	8.80 ± 2.56	<0.001
BPC (×10^9/L)	182.71 ± 51.40	217.02 ± 59.12	0.001
Neutrophil (%)	60.46 ± 9.31	69.59 ± 12.48	<0.001
Potassium (mM)	3.69 ± 0.42	3.80 ± 0.37	0.122
Sodium (mM)	142.68 ± 2.02	140.51 ± 2.86	<0.001
NT‐proBNP (pg/mL)	35.75–252.10 (63.20)	325.13–1832.75 (868.85)	<0.001
hs‐cTnI (ng/mL)	0.010–0.021 (0.010)	2.861–68.956 (29.367)	<0.001
Myoglobin (ng/mL)	16.20–28.60 (21.30)	40.90–331.30 (125.40)	<0.001
CK‐MB (ng/mL)	1.00–2.03 (1.30)	12.60–236.80 (101.30)	<0.001
d‐dimer (mg/L)	0.19–0.39 (0.25)	0.19–0.64 (0.31)	0.376
CRP (mg/dL)	0.20–3.19 (1.49)	0.89–5.08 (2.37)	0.486
TB (μmol/L)	13.69 ± 6.22	16.56 ± 7.15	0.020
DB (μmol/L)	1.55–3.50 (2.60)	1.80–3.80 (2.80)	0.665
TC (mmol/L)	4.25 ± 1.04	4.65 ± 1.32	0.073
TG (mmol/L)	1.02–1.98 (1.22)	0.96–1.840 (1.25)	0.951
HDL‐C (mM)	1.05 ± 0.24	1.02 ± 0.21	0.568
LDL‐C (mM)	2.86 ± 0.81	3.23 ± 1.08	0.038
TP (g/L)	66.64 ± 5.16	66.58 ± 6.49	0.957
ALB (g/L)	40.20 ± 3.58	38.75 ± 4.00	0.037
ALT (U/L)	13.00–23.00 (17.50)	21.00–45.00 (32.00)	0.063
AST (U/L)	16.00–26.75 (19.00)	25.00–150.00 (58.00)	<0.001
CK (ng/mL)	54.25–105.25 (76.50)	99.00–1242.00 (448.00)	<0.001
BUN (mM)	4.75–6.48 (5.30)	4.27–6.46 (5.52)	0.548
Creatinine (μM)	67.25–87.00 (75.50)	68.00–92.00 (81.00)	0.115
Uric acid (μM)	374.79 ± 111.53	382.84 ± 120.11	0.701

*Note*: All data were presented as an absolute number (percentage), the mean (standard deviation), or the 25th–75th percentile (median).

Abbreviations: ALB, albumin; ALT, alanine amino transferase; AST, aspartate amino transferase; BMI, body mass index; BPC, blood platelet cell; BUN, blood urea nitrogen; CK, creatine kinase; CRP, C‐reactive protein; DB, direct bilirubin; DBP, diastolic blood pressure; HDL‐C, high‐density lipoprotein; LDL‐C, low density lipoprotein; NT‐proBNP, N‐terminal pro‐B‐type natriuretic peptide; RBC, red blood cell; SBP, systolic blood pressure; TB, total bilirubin; TC, total cholesterol; TG, triglyceride; TP, total protein; WBC, white blood cell.

In the validation cohort, we also tested the importance of features, the results show in all cohorts, cZNF292 was the most important feature (Figure S6A–C). These results further confirmed the importance of cZNF292 for the diagnosis of AMI.

Besides, in all patients, including the discovery and validation cohorts, we separated the patients by using mean value levels of blood cZNF292. We found that patients with high‐level blood cZNF292 had lower sodium, and higher WBC, neutrophil (%), NT‐proBNP, hs‐cTnI, myoglobin, CK‐MB, TB, AST, CK, and creatinine, suggesting that a high‐level blood cZNF292 is likely to have a similar pattern with AMI (Table S6). Correlation analysis also showed that there was no strong correlation between blood cZNF292 and clinical and experimental variables (Table S7), suggesting blood cZNF292 as a distinct factor.

These results suggest that a prediction model “CM + cZNF292” has been constructed for AMI.

### 2.5 Functions of cZNF292 in endothelial cells and cardiomyocytes

**FIGURE 5:**
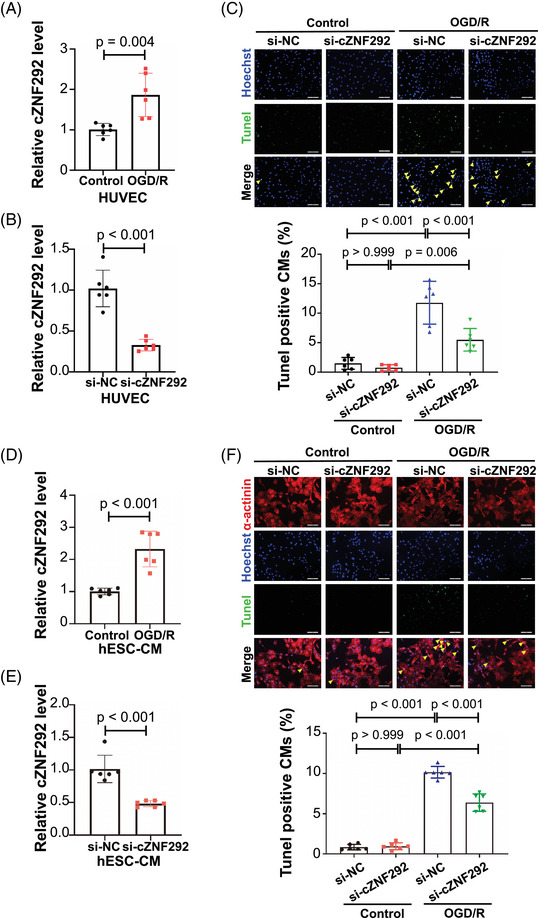
siRNA‐mediated silencing of cZNF292 inhibits apoptosis in human umbilical vein endothelial cell (HUVEC) and human embryonic stem cell–derived cardiomyocyte (hESC‐CM): (A) q‐polymerase chain reaction (PCR) analysis of cZNF292 in HUVEC treated with oxygen glucose deprivation/re‐oxygenation (OGD/R) (*n* = 6/group); (B) qPCR analysis of cZNF292 in HUVEC treated with siRNA cZNF292 (*n* = 6/group); (C) the TUNEL staining analysis of HUVEC apoptosis in OGD/R model treated with cZNF292 siRNA (*n* = 6/group); (D) qPCR analysis of cZNF292 in hESC‐CM treated with OGD/R (*n* = 6/group); (E) qPCR analysis of cZNF292 in hESC‐CM treated with siRNA cZNF292 (*n* = 6/group); (F) the TUNEL staining analysis of hESC‐CM apoptosis in OGD/R model treated with cZNF292 siRNA (*n* = 6/group). Scale bar: 100 μm in parts (C) and (F).

As cZNF292 has been indicated to potentially regulate apoptosis,^27^ we investigated its function and regulation in endothelial cells and cardiomyocytes treated by oxygen glucose deprivation/reoxygenation (OGD/R). The cZNF292 level was significantly increased in human umbilical vein endothelial cells (HUVECs) treated by OGD/R (Figure 5A). Knockdown of cZNF292 could attenuate the apoptosis in HUVECs treated by OGD/R (Figure 5B,C). Similarly, in human embryonic stem cell–derived cardiomyocytes (hESC‐CMs) treated by OGD/R, we also found that cZNF292 was significantly increased (Figure 5D) and the knockdown of cZNF292 could attenuate the apoptosis (Figure 5E,F). Collectively, these data confirm that the knockdown of cZNF292 can decrease the apoptosis of endothelial cells and cardiomyocytes in OGD/R.

## 3 DISCUSSION

Aiming at investigating if combining circRNAs biomarkers with an artificial intelligence approach could be used to diagnose CVD, we used AMI as a model setup to prove the claim. In this study, circulating cZNF292 has been identified as a potential biomarker for AMI, and a prediction model “CM + cZNF292” was constructed based on an artificial intelligence approach, demonstrating feasibility for our hypothesis.

The unique features as below made circRNAs in the circulation as promising biomarkers.^14^, ^18^ First, the circularized structure of circRNAs protects them from exonuclease activities, making them stable as biomarkers.^10^ Second, circRNAs can be easily detected in blood, serum, and plasma.^14^, ^18^ Third, some circRNAs are highly conserved and expressed in a tissue, cell, and developmental stage‐specific manner or response to disease distinct.^10^, ^14^ In our study, we found that cZNF292 is a stable molecule as demonstrated by resistance to be incubated at room temperature for 24 h or subjected to five cycles of freezing and thawing. Besides, we found that among the five hypoxia‐induced circRNAs we determined, all circRNAs expect cTSHSD1 can be detected in whole blood. Furthermore, cAFF1 and cSRSF4 were detected in serum and plasma, whereas the rest were not. Interestingly, we found cZNF292 to be not elevated in peripheral blood mononuclear cells of patients with ischemic cardiomyopathy or CAD indicating a vascular origin for the measured increase in circulating circRNA in AMI (Figure S7). Thus, we choose whole blood to detect the four hypoxia‐induced circRNAs, including cZNF292, cAFF1, cDENND4C, and cSRSF4. Interestingly, a recent study has determined circRNAs as biomarkers in AMI, and they found that cardiac‐enriched circRNAs had poor detectability in plasma and serum,^28^ which is consistent with our data.

Some circulating biomarkers for CVDs have been highlighted.^15^, ^16^, ^17^, ^18^ For example, the circRNA circ_0062960 was found to be increased in plasma in heart failure patients, with an area under the ROC curve of 0.838 for the diagnosis of heart failure.^29^ In addition, serum circRNAs DNAJC6, TMEM56, and MBOAT2 were decreased in hypertrophic obstructive cardiomyopathy patients.^16^ Interestingly, plasma circ_0003258, circ_0051238, circ_0051239, and circ_0089762 can be used for categorizing etiologies of dilated cardiomyopathy.^30^ Moreover, plasma circ_0001445 was associated with higher coronary atherosclerosis extent and severity.^31^ In this study, we screened the five hypoxia‐induced circRNAs in endothelial cells and identified that cZNF292 in whole blood could be a potential biomarker for AMI. As a circRNA expressed in hypoxic conditions,^21^ cZNF292 inhibition has been reported to attenuate proliferation, radioresistance and vasculogenic mimicry of hypoxic hepatoma cells,^32^ and oxygen glucose deprivation‐/reperfusion‐induced rat neural stem cells apoptosis.^27^ Besides, cZNF292 knockdown can also suppress human glioma tube formation.^33^ Very recently, a locus‐conserved cirRNA cZNF292 has been reported to control endothelial cell flow response.^34^ Here, we report for the first time that the circulating circRNA cZNF292 could be a potential biomarker for AMI. The function of cZNF292 in cardiac diseases is unknown. In a cellular OGD/R model, we demonstrated that the knockdown of cZNF292 could decrease the apoptosis of endothelial cells and cardiomyocytes, suggesting that it might also play a functional role in AMI.

Unlike the conventional statistical model that constructs relationships between variables in a mostly linear manner, machine learning can provide optimal predictions as it can identify relations in an interpretable way.^23^ From a clinical point of view, machine learning is a new strategy to identify biomarkers in individuals.^35^ The present study is a proof‐of‐concept study to determine if combining circRNAs with an artificial intelligence approach works in diagnosing CVD and AMI was used a model setup to prove the claim. In this work, we automatically selected features for accurately diagnosing AMI in human based on lasso, which is one of the widely used machine learning. Based on our model, we can diagnose AMI by using basic CM and the expression level of cZNF292, providing a novel way for studying AMI, and this method can also be applied in other fields.

Several limitations of the present study should be highlighted. First, the normalization of extracellular RNAs in biofluid is an issue in this field.^36^, ^37^ As some longer RNAs (e.g., GAPDH mRNA) cannot be ruled out with regards to the changes with CVD, we used a spike‐in, control miRNA (cel‐miR‐39) for normalizing.^36^, ^37^ An internal control of circulating circRNA will be required in the future. Second, circulating circRNAs are present in exosomes.^38^ We have not detected if cZNF292 could be detected in exosomes. Nevertheless, for the identification of biomarkers, it will be more ideal to detect them in blood directly as the isolation of exosomes from the blood needs ultracentrifugation and more amount of blood.

## 4 CONCLUSIONS

In this section, we identify circulating circRNA cZNF292 as a potential biomarker for AMI and construct a prediction model “CM + cZNF292,” which only needs the information of basic CM, including age, gender, BMI, heart rate, and DBP. Although the result of this medium study needs to be validated by independent larger cohorts, our study indicates a potential opportunity to improve clinical assessment and guide clinical decision‐making by using a model based on easily available clinical data and circulating circRNAs. Combining circRNA biomarkers with an artificial intelligence approach could be used to diagnose CVD.

## 5 MATERIALS AND METHODS

### 5.1 Patients

All human investigations conformed to the principles outlined in the Declaration of Helsinki and were approved by the institutional review committees of Tongji Hospital or Shanghai Tenth's People Hospital, Tongji University (2014‐002 and 22KT64). All patients were recruited with written informed consent. In the discovery cohort, the MI and non‐MI patients were confirmed by coronary angiography.

### 5.2 Blood processing

The samples were divided into three categories: whole blood, plasma, and serum. For whole blood and plasma collection, venous blood was collected in vacuum anticoagulant (EDTA‐K2) tubes. After blood collection, whole blood samples were immediately reversed and mixed and then stored at −80°C. After centrifuged at 4°C for 5 min at 3000 rpm, the plasma samples were collected in RNase/DNase‐free tubes and then stored at −80°C. For serum collection, venous blood was collected in vacuum coagulant tubes. After that, blood samples were centrifuged at 4°C for 15 min at 3000 rpm, and serum samples were collected into RNase/DNase‐free tubes and immediately aliquoted and frozen at −80°C.

### 5.3 RNA isolation

The total RNA was isolated from the blood samples using mirVana miRNA Isolation Kit (Thermo Scientific#AM1561) according to the manufacturer's instructions. Blood samples (whole blood, plasma, and serum) were removed from −80°C in advance and melted on ice. After thawing, 300 μL of blood sample was transferred to RNase/DNase‐free tubes and used to extract the total RNA. Caenorhabditis elegans miR‐39 (cel‐miR‐39) of 50 pmol/L was added as the spike‐in control after an equal volume of Lysis/Binding solution (300 μL) was added. The blood sample was incubated for 10 min on ice after 1/10 volume of miRNA Homogenate Additive was added. Then, equal to the initial lysate volume (before the addition of the miRNA Homogenate Additive) of acid‐phenol: chloroform was added. After centrifugation (12,000*g* for 1 min at 4°C), the supernatant was transferred to fresh RNase/DNase‐free tubes. Overall, 100% ethanol (1.25 volume of the supernatant) was added to supernatant and transferred to the Filter Cartridge. The filter cartridges were centrifuged (12,000*g* for 1 min at 4°C) and then washed by 700 μL Wash solution 1 once and 500 μL Wash solution 2/3 twice. Finally, each sample was eluted in 100 μL of DEPC‐treated water (95°C).

### 5.4 Quantification of CircRNA

Total RNA was reverse transcribed using the iScript cDNA Synthesis Kit (Bio‐Rad) according to the manufacturer's instructions. For cDNA synthesis, 14 μL RNA was reverse‐transcribed into 20 μL cDNA using 4 μL 5 × iScript reaction mix, 1 μL iScript reverse transcriptase, and 0.25 μM miRNA‐specific RT primer (RiboBio). The samples were incubated for 5 min at 25°C, 10 min at 37°C, and 5 min at 85°C.

Quantification of circRNA was performed by quantitative reverse‐transcription polymerase chain reactions (qRT‐PCRs) using Takara SYBR Premix Ex Taq (Tli RNase H Plus) in the LightCycler 480 II (Roche). For the detection of the expression of circRNAs, the 4 μL cDNA diluted with nuclease‐free water (1:20) was amplified with 0.5 μM primers in TB Green Premix Ex Taq (TaKaRa) at a reaction volume of 10 μL. The qPCR was performed using the following protocol: 95°C for 5 min, followed by 40 cycles with 95°C for 15 s, 55–60°C for 30 s, 72°C for 30 s, finally 95°C for 15 s, 60°C for 60 s, and 95°C for 15 s. The expression level of circRNAs in the blood sample was normalized using spike‐in cel‐miR‐39. All qRT‐PCR reactions were performed in triplicate, and the signal was collected at the end of every cycle. The relative expression level was calculated using the 2^−ΔΔ^ *^Ct^* method. The sequences for qRT‐PCR primers were listed in Table S8.

### 5.5 Detection of CircRNAs

Total RNA was reverse‐transcribed with the iScript cDNA Synthesis Kit (Bio‐Rad) according to the manufacturer's instructions. Then, circRNAs were detected by semiquantitative PCR using the KOD‐Plus‐Neo Kit (TOYOBO). Finally, the PCR products were electrophoresed on 2%–3% agarose gel and visualized under UV light. The sequences for semiquantitative PCR primers were the same as qRT‐PCR.

### 5.6 CircRNA stability assay

RNA of the human whole blood of healthy persons was isolated from three ways: directly isolated, isolated after 24 h at room temperature (24 h RT), and five freeze/thaw cycles (5× thaw/freeze). For directly isolated, blood samples were directly isolated after thawed on ice. In parallel, blood samples were incubated for 24 h at room temperature or sequentially frozen for 1 h and then thawed on ice five times. The next day, RNA was isolated as previously mentioned. Mean CT value of circRNA cZNF292 and ZNF292 was detected by qRT‐PCR and semiquantitative PCR as previously mentioned. Finally, the PCR products were electrophoresed on 2%–3% agarose gel and visualized under UV light. The sequences for qRT‐PCR primers are listed in Table S8.

### 5.7 RNase R treatment

A volume of 0.5 mg RNA was incubated for 10 min at 37°C with or without 0.5 units of RNase R. Mean CT value of cZNF292 and ZNF292 was detected by qRT‐PCR as previously mentioned.

### 5.8 Feature selection and training

Lasso as one of the most widely used methods in statistics and machine learning is commonly used for feature selection.^24^ In this work, all the feature selection was performed based on Lasso. Assume *x_i_* = (*x_i_*_1_, *x_i_*_2_,…,*x_ip_*) were the predictor variables, where *p* was the number of predictor variables, and *y_i_* were response variables, *i* = 1,2,…,*n*, *n* was the number of samples. The goal of the algorithm was to minimize

(1) $$\sum_{i=1}^{n}{\left(y_{i}-\sum_{j=1}^{p}x_{ij}\beta_{j}\right)}^{2}+\lambda \sum_{j=1}^{p}\left|\beta_{j}\right|$$

where *λ* was the tuning parameter. Letting $\hat{\beta }=\left(\beta_{1},\text{…},\beta_{p}\right)$ be the estimated parameters. We constructed the Lasso fit using 10‐fold cross validation, and $\hat{\beta }$ were identified by selecting the *λ* with minimum mean squared error. Then if *β_i_* > 0, we selected the *i*th variable as one of the prediction features.

In our data, we have eight features, which were circRNAs, including cZNF292, cSRSF4, and CM, including age, gender, BMI, heart rate, SBP, and DBP. We selected six variables as features based on the Lasso, which were cZNF292, age, gender, BMI, heart rate, and DBP. We used CM + cZNF292 to indicate the six selected features, where CM means a clinical model, including age, gender, BMI, heart rate, and DBP.

### 5.9 Cell culture and treatments

HUVECs were maintained in DMEM (Corning, USA) with 4.5 g/L glucose supplemented with 10% fetal bovine serum (Biological Industries, Israel) and 1% Penicillin‐Streptomycin Solution (KeyGEN BioTECH, China) at 37°C in 5% CO_2_. The apoptosis of HUVEC was detected using the TUNEL FITC Apoptosis Detection Kit (Vazyme, China) according to the manufacturer's instructions. Briefly, HUVEC were fixed with 4% paraformaldehyde, permeabilized with 0.5% Triton X‐100, and then blocked with 5% bovine serum albumin for 1 h at room temperature. Next, HUVEC were stained with the TUNEL FITC Apoptosis Detection Kit (Vazyme, China) according to the manufacturer's instructions, and the nuclei were stained with Hoechst. A confocal microscope (Leica, Germany) was used to photograph, and at least 10 fields/holes (200× magnification) were analyzed. The apoptosis of HUVEC was evaluated by calculating the percentage of TUNEL‐positive nuclei.

The hESC line H9 was cultured using E8 medium (A1517001, Thermo Fisher, USA) on Matrigel (354230, Corning, USA)‐coated 6‐well plates. When hESCs grow to 80% confluence, cells were digested using Versene (15040‐066, Life Technologies, USA) and passaged into 12‐well plates. Differentiation for cardiac cells begins when hESCs grow to 80% confluence. On days 0–2 of differentiation, hESCs were cultured in RPMI1640 medium (72400047, Life Technologies, USA) with 4 μM CHIR99021 (S1263, Selleck, USA), 0.5 mg/mL human recombinant albumin (A9731, Sigma, USA), and 0.2 mg/mL sodium vitamin C phosphate (A8960, Sigma, USA). On days 2–4 of differentiation, hESCs were cultured in RPMI1640 medium with 5 μM IWP‐2 (S7085, Selleck, USA), 0.5 mg/mL human recombinant albumin, and 0.2 mg/mL sodium vitamin C phosphate. On days 4–6 of differentiation, cells were cultured in RPMI1640 medium with 0.5 mg/mL human recombinant albumin and 0.2 mg/mL sodium vitamin C phosphate to terminate the induction of small molecules. From day 8 of differentiation, cells were cultured in RPMI1640 medium containing B27 (17504‐044, Thermo Fisher, USA). By day 14 of differentiation, the cells were passaged into 12‐well plates at 1:4 ratio. Purification of cardiomyocytes begins after day 15, and the purification of cardiomyocytes is performed using RPMI1640 medium without d‐glucose (11879020, Life Technologies, USA) with DL‐lactic acid (L4263, Sigma, USA), 0.5 mg/mL human recombinant albumin, and 0.2 mg/mL sodium vitamin C phosphate. Cardiomyocytes with high purity can be obtained at the end of metabolic screening, and cardiomyocytes matured to days 30–60 can be used for cell experiments.

To construct the OGD/R model, HUVEC or hESC‐CM were cultured with serum‐free DMEM (no glucose) in an airtight chamber with an Anaero pack containing 5% CO2 and 95% N2 at 37°C. After 6 h, cells were transferred to a normal incubator and cultured in DMEM containing serum and glucose for recovery 12 h. For cZNF292 suppression, si‐cZNF292 (100 nM) was incubated for 48 h using Lipofectamine 2000 (Invitrogen, USA). The siRNA targeting human cZNF292 was 5′‐CAGAACACACACUAUAGAG‐3′.

### 5.10 Statistical analysis

The statistical results were analyzed with SPSS 20.0 and presented as mean ± standard deviation was plotted with GraphPad Prism 8.0. An independent sample *t* test was used for comparison between the two groups, and one‐way ANOVA test or two‐way ANOVA followed by Bonferroni's multiple comparisons test was used for comparison among the three groups and above. Correlation analyses were analyzed using Spearman's method. ROC curves were created by MedCale. Two ROC curves were compared by the DeLong test. A value of *p* less than 0.05 was considered statistically significant.

## AUTHOR CONTRIBUTIONS

*Designed the study; instructed all experiments; and drafted the manuscript*: Jiahong Xu, Stefanie Dimmeler. *Performed the experiments and analyzed the data*: Qiulian Zhou, Jes‐Niels Boeckel, Yuzheng Bai, Yicheng Lv, Meiyu Hu, and Danni Meng. *Collected and processed sample*: Jianhua Yao, Yuan Xie, Pujiao Yu, Peng Xi, Jiahong Xu, and Yi Zhang. *Performed feature selection and training and analyzed the data*: Juan Zhao. *Revised the manuscript*: Qiulian Zhou, Jes‐Niels Boeckel, and Juan Zhao. Qiulian Zhou, Jes‐Niels Boeckel, Jianhua Yao and Juan Zhao contributed equally to this work. All authors have read and approved the final manuscript.

## CONFLICT OF INTEREST STATEMENT

Authors have no conflict of interests to disclose.

## ETHICS STATEMENT

All human investigations conformed to the principles outlined in the Declaration of Helsinki and were approved by the institutional review committees of Tongji Hospital or Shanghai Tenth's People Hospital, Tongji University (2014‐002 and 22KT64). All patients were recruited with written informed consent.

## Supporting information

Supporting Information

## Data Availability

The data for this study are available by contacting the corresponding author upon reasonable request.
