# Supplementary material for: Diagnosis of acute myocardial infarction using a combination of circulating circular RNA cZNF292 and clinical information based on machine learning
Source: MedComm (2020). 2023 Jun 13;4(3):e299. doi: 10.1002/mco2.299 (PMC10264944; doi:10.1002/mco2.299)
Supplement: Supplementary file 1 — Supporting Information [file MCO2-4-e299-s001.docx]

**Supplementary Materials**

**Diagnosis of** **acute myocardial infarction using a combination of circulating circular RNA cZNF292 and clinical information based on machine learning**

Qiulian Zhou^1,2,#^, Jes-Niels Boeckel^3,4#^, Jianhua Yao^5,6, #^, Juan Zhao^1,2,7,#^, Yuzheng Bai^1^, Yicheng Lv^1^, Meiyu Hu^1^, Danni Meng^1^, Yuan Xie^8^, Pujiao Yu^8^, Peng Xi^8^, Jiahong Xu^8^, Yi Zhang^5^, Stefanie Dimmeler^3,*^, Junjie Xiao^1,2,*^

**Supplemental Figures**Figure S1. Validation of expression of selected circRNAs in whole blood, plasma and serum by semiquantitative PCR.

Figure S2. Validation of expression of selected circRNAs in whole blood, plasma and serum by qRT-PCR.

Figure S3. Expression of cZNF292 in cardiovascular compartments.

Figure S4. 10-fold cross validation plot for the parameter lambda of the discovery cohort.

Figure S5. ZNF292 mRNA is unstable in human whole blood.

Figure S6. The Feature importance test based on Bagging Decision Trees for validation cohorts.

Figure S7. Expression of cZNF292 in the blood of patients with ischemic cardiomyopathy or coronary artery disease.

**Supplemental Tables**Table S1. Lasso parameters results.

Table S2. ROC results in the training cohort.
Table S3. ROC results for predicting AMI and non-AMI.

Table S4. ROC results for predicting UA and AMI.
Table S5. ROC results for predicting ACS and non-ACS.
Table S6. The characteristics of all patients with different levels of cZNF292.

Table S7. Correlation analysis between blood cZNF292 and clinical experimental variables.
Table S8. The sequences of primers used for RT-PCR and semiquantitative PCR.

**
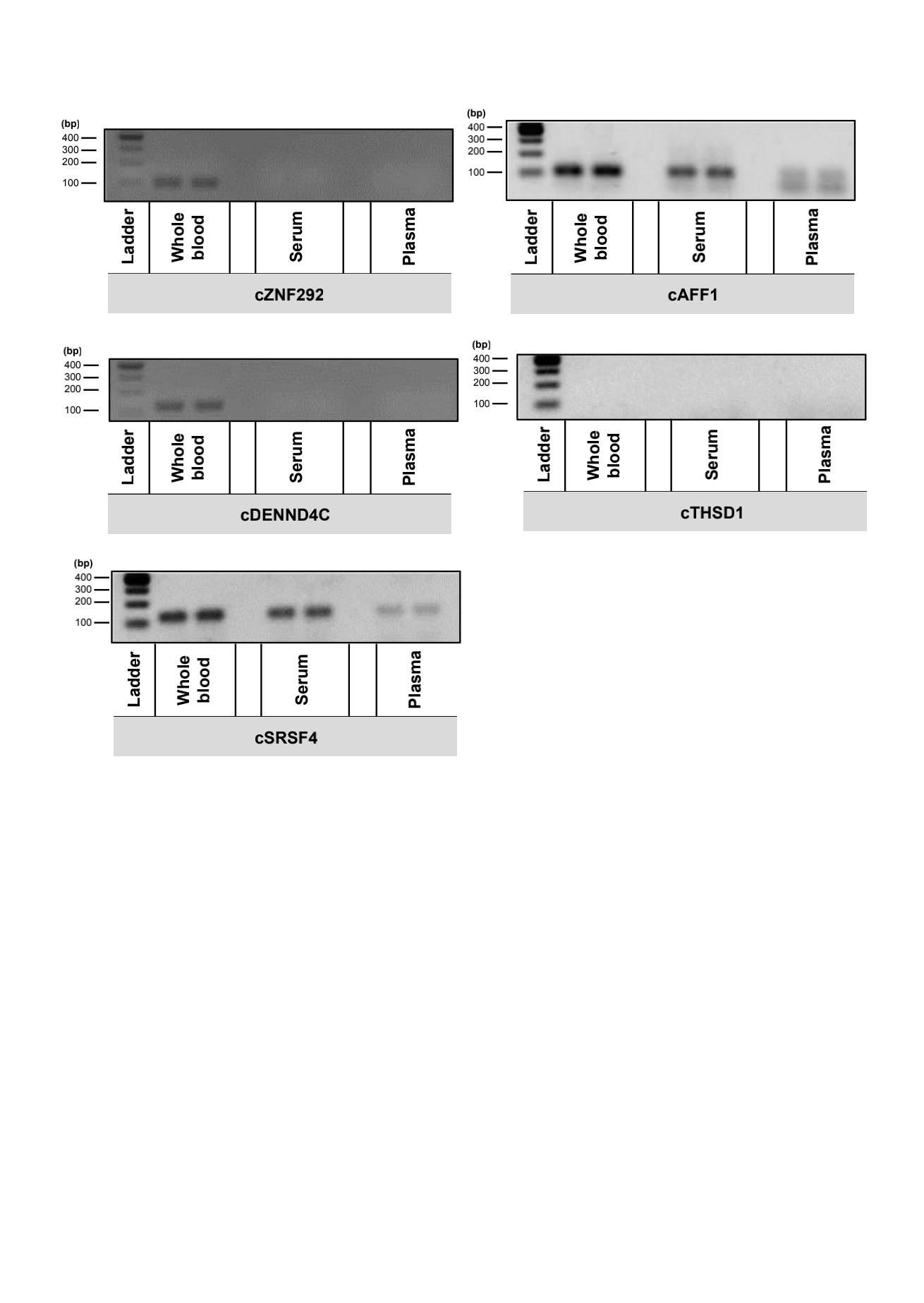
**

**Figure S1. Validation of expression of selected circRNAs in whole blood, plasma and serum by semiquantitative PCR.**

Semiquantitative PCR analysis of circRNAs including cZNF292, cAFF1, cDENND4C, cTHSD1, and cSRSF4 in human whole blood, plasma and serum.

**
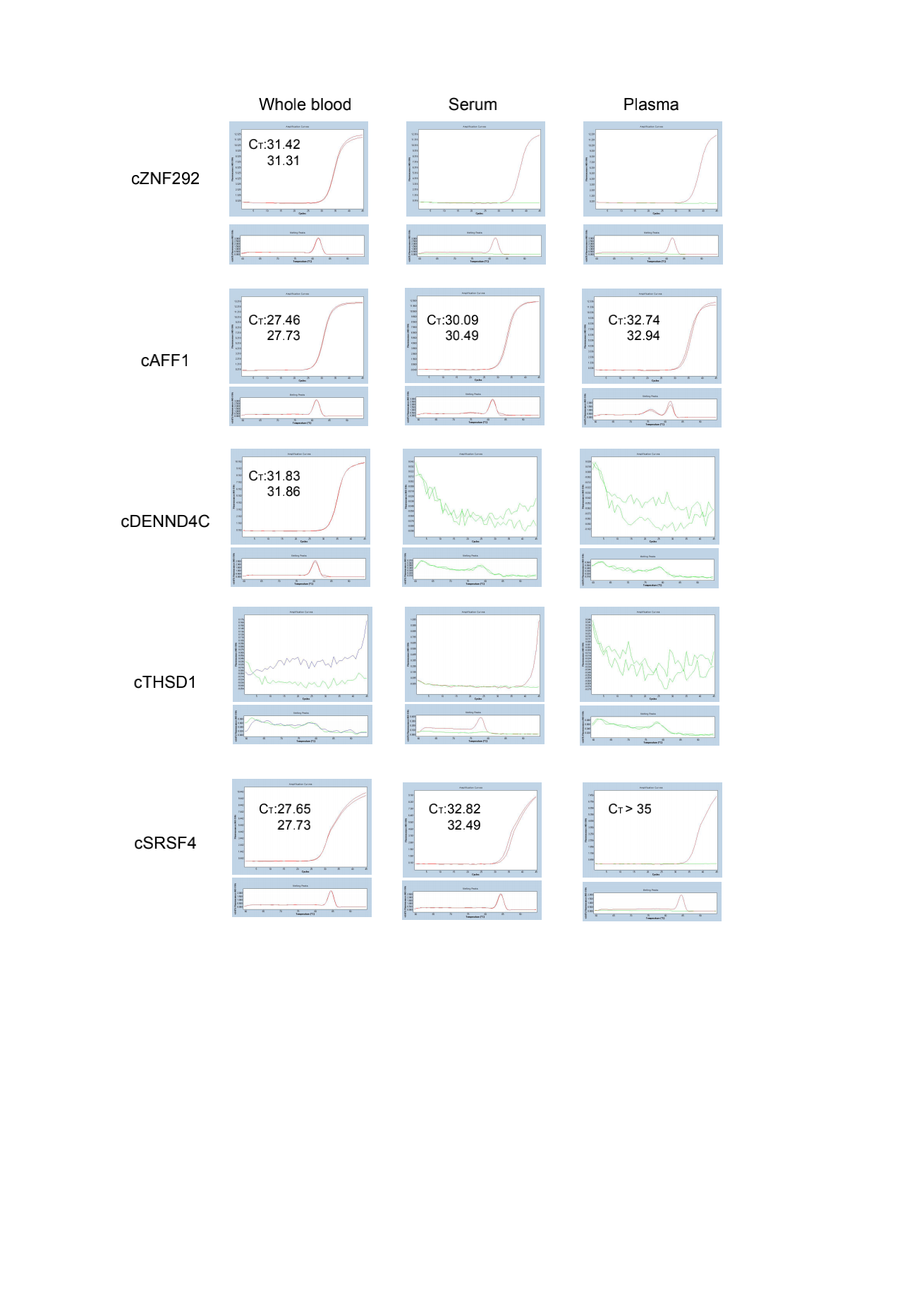
**

**Figure S2. Validation of expression of selected circRNAs in whole blood, plasma and serum by qRT-PCR.**

The qPCR amplification curves and dissolution curve of circRNAs including cZNF292, cAFF1, cDENND4C, cTHSD1, and cSRSF4 in whole blood, plasma and serum.

**
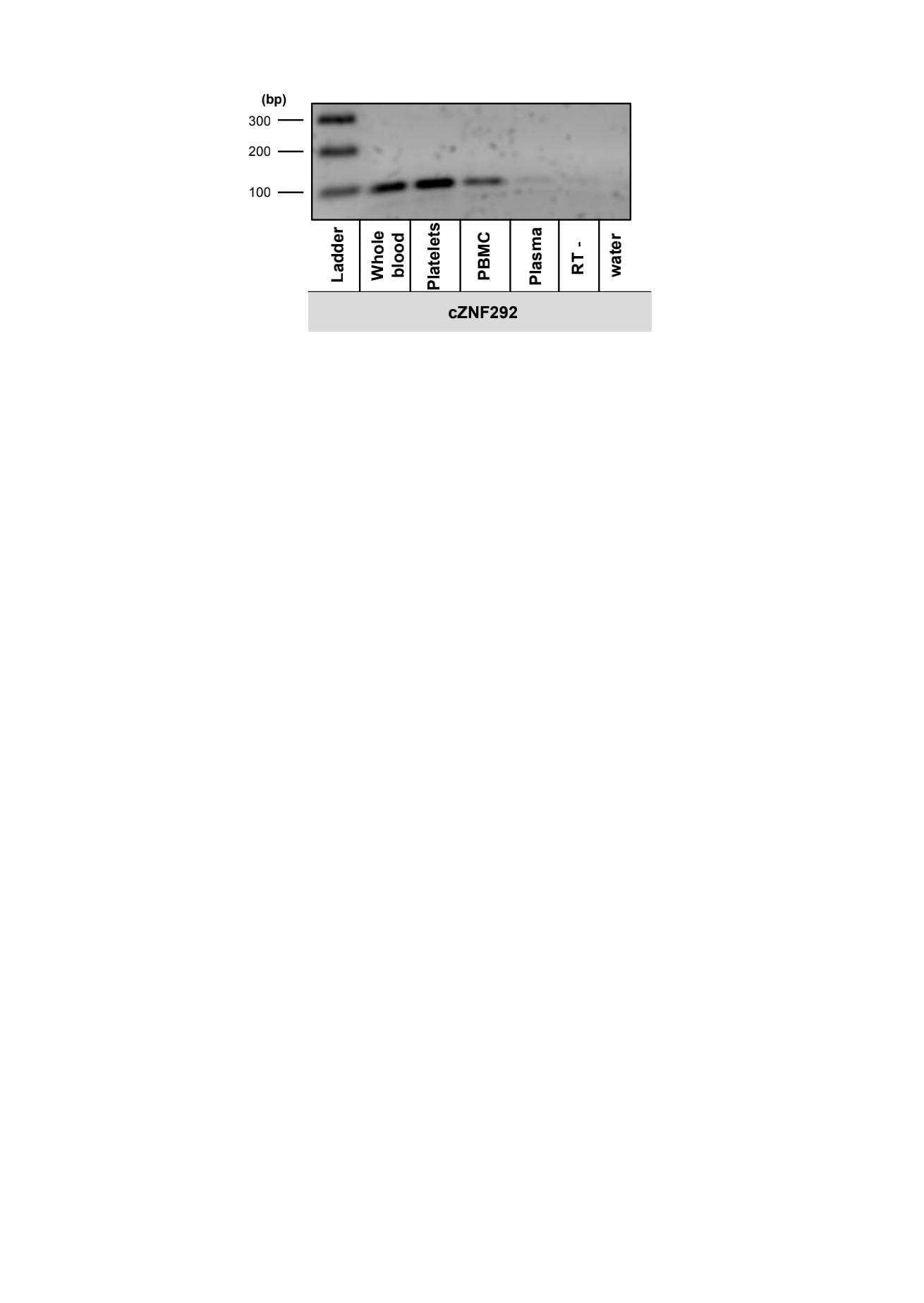
**

**Figure S3. Expression of cZNF292 in cardiovascular compartments.**

cZNF292 was detected using qPCR with subsequent agarose gel electrophoresis using divergent-orientated primers. RNA was isolated from whole blood, platelets, peripheral blood mononuclear cells (PBMCs) and plasma with subsequent DNA digestion. Specificity of the reaction was assessed by adding negative control samples without reverse transcription reaction or only using water as template for reverse transcription.

**
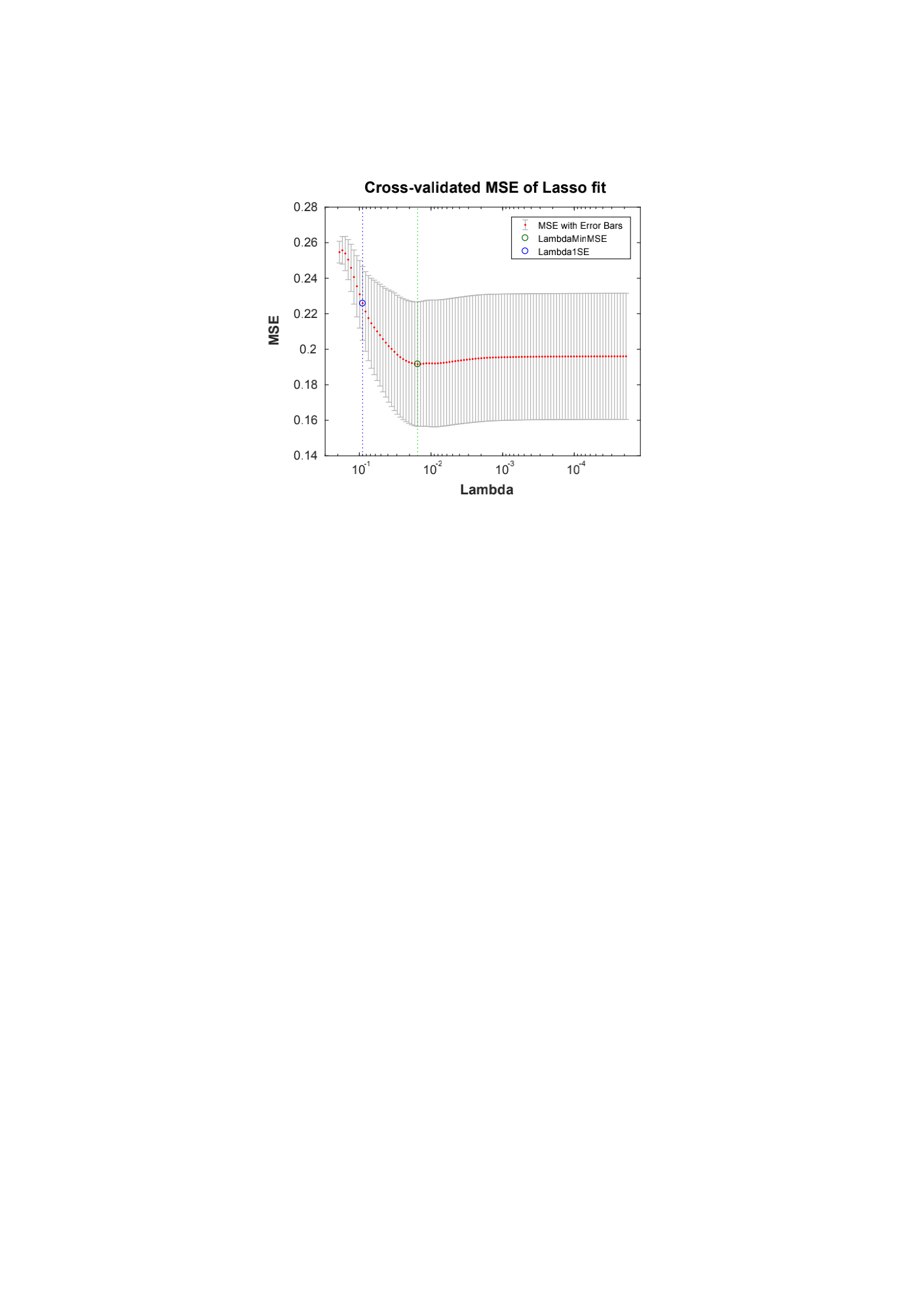
**

**Figure S4. 10-fold cross validation plot for the parameter lambda of the discovery cohort.**

The X-coordinate was the tuning parameter lambda. The Y-coordinate was the mean standard error (MSE). We selected the lambda when the model has minimal MSE.

**
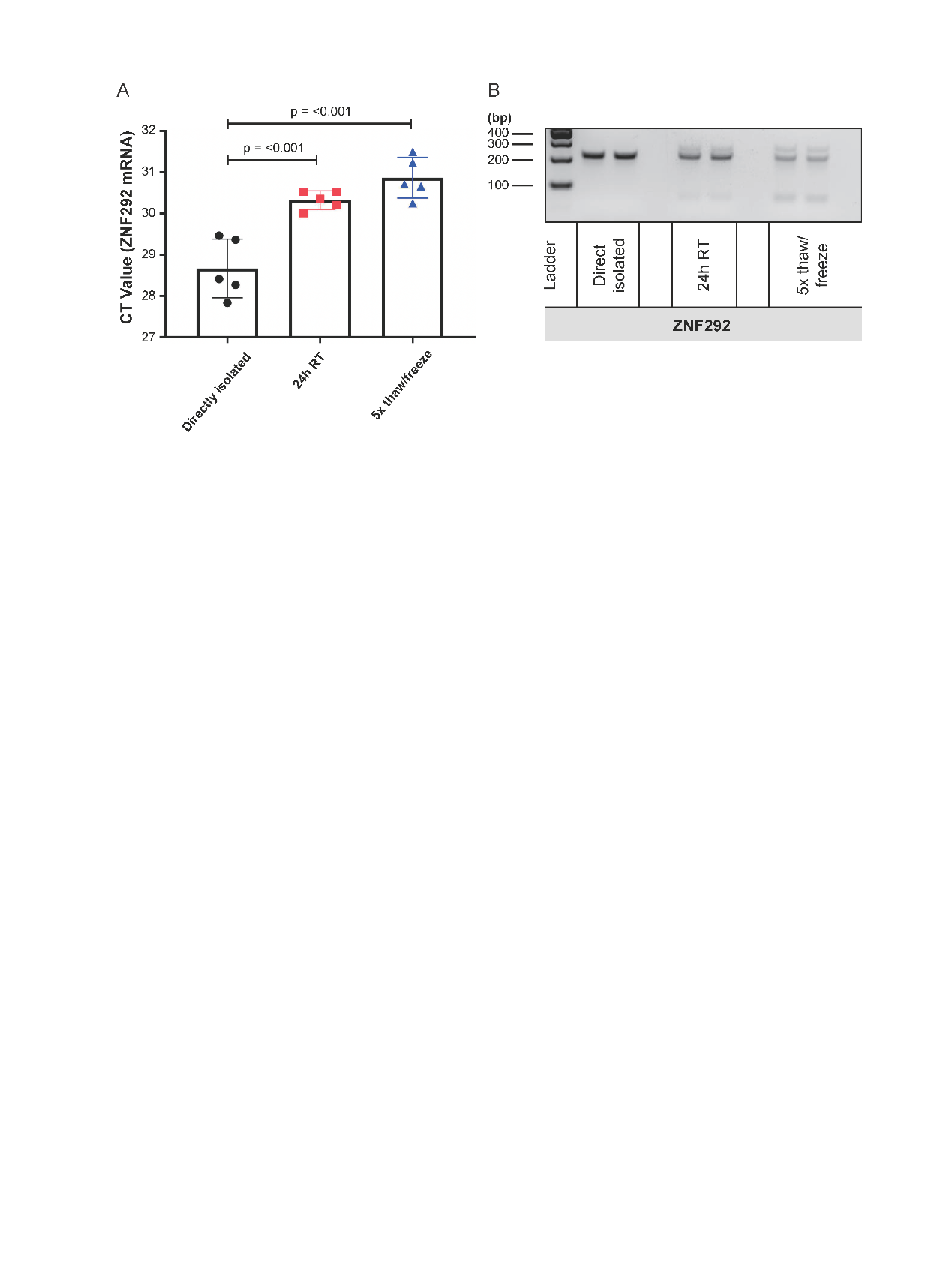
**

**Figure S5. ZNF292 mRNA is unstable in human whole blood.**

(A) The mean CT value of ZNF292 in human whole blood treated with directly isolated, incubated for 24 hours at RT or frozen and thawed for five cycles before isolating RNA (n=5/group). (B) Semiquantitative PCR analysis of ZNF292 in human whole blood.

**
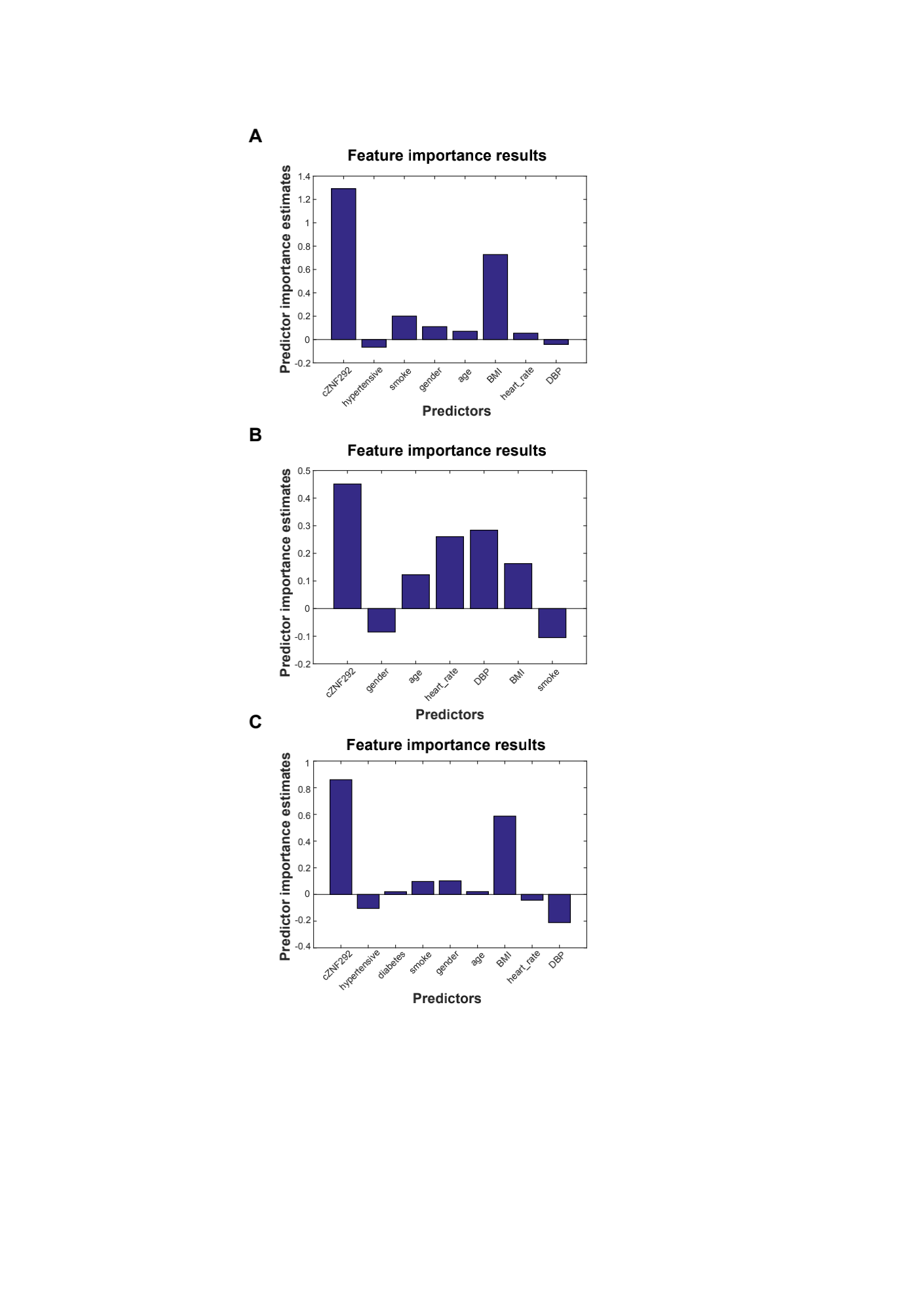
**

**Figure S6. The Feature importance test based on Bagging Decision Trees for validation cohorts.**

(A) The feature importance test for AMI and non-AMI (here refers to unstable angina or stable angina) patients. AMI, acute myocardial infarction. (B) The feature importance test for AMI and unstable angina (UA) patients. (C) The feature importance test for ACS and non-ACS patients. ACS, acute coronary syndrome (including unstable angina and AMI).

**
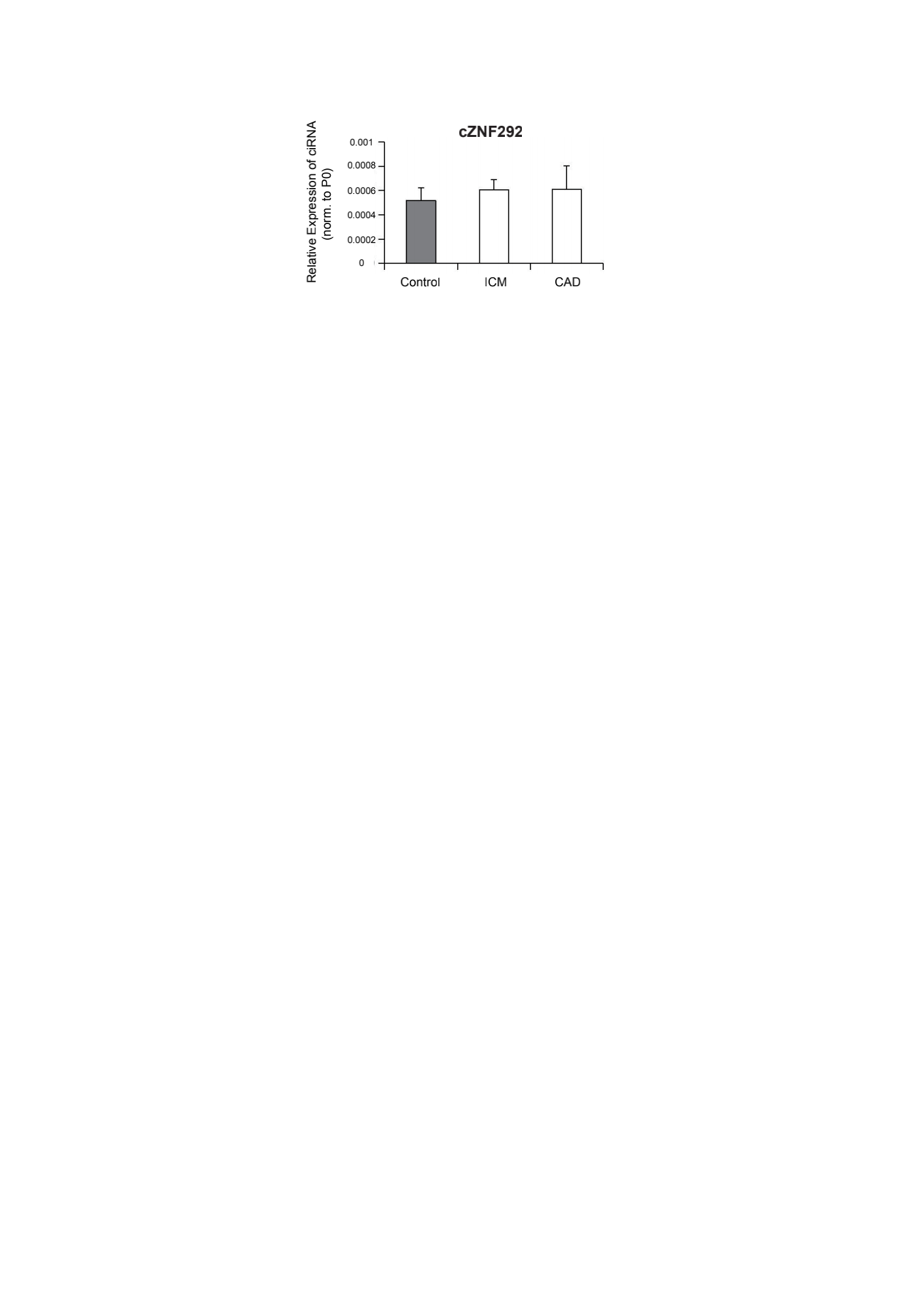
**

**Figure S7. Expression of cZNF292 in the blood of patients with ischemic cardiomyopathy or coronary artery disease.**

cZNF292 was detected in PBMNCs of patients with ischemic cardiomyopathy or coronary artery disease using qPCR with divergent-orientated primers compared to non-diseased controls (n=4/group). RNA was isolated from peripheral blood mononuclear cells (PBMCs) with subsequent DNA digestion. Specificity of the reaction was assessed by adding negative control samples without reverse transcription reaction or only using water as template for reverse transcription.

**Table S1. Lasso parameters results.**

| **Variables** | B |
| --- | --- |
| **cZNF292** | 0.057591477 |
| **cSRSF4** | 0 |
| **age** | 0.014646872 |
| **BMI** | -0.041342236 |
| **gender** | 0.149467395 |
| **heart-rate** | -0.002830049 |
| **systolic blood pressure** | 0 |
| **diastolic blood pressure** | 0.007544739 |

Notes:B is the training results for beta, the variables with non-zero B were selected as predictors.

**Table S2. ROC results in the training cohort.**

| **Predictors** | **AUC** | **95%CI** | |
| --- | --- | --- | --- |
| cZNF292 | 0.747 | 0.619 | 0.852 |
| cSRSF4 | 0.672 | 0.541 | 0.78 |
| age | 0.714 | 0.583 | 0.822 |
| BMI | 0.641 | 0.495 | 0.754 |
| gender | 0.551 | 0.437 | 0.648 |
| Heart_rate | 0.536 | 0.405 | 0.667 |
| SBP | 0.568 | 0.441 | 0.695 |
| DBP | 0.64 | 0.489 | 0.747 |
| CM+cZNF292 | 0.871 | 0.763 | 0.94 |
| CM+cSRSF4 | 0.852 | 0.74 | 0.929 |
| cZNF292+age | 0.789 | 0.661 | 0.884 |
| cZNF292+BMI | 0.817 | 0.699 | 0.905 |
| cZNF292+gender | 0.748 | 0.623 | 0.85 |
| cZNF292+Heart_rate | 0.733 | 0.594 | 0.829 |
| cZNF292+SBP | 0.781 | 0.649 | 0.871 |
| cZNF292+DBP | 0.783 | 0.654 | 0.874 |

**Table S3. ROC results for predicting AMI and non-AMI.**

| **Predictors** | **AUC** | **95%CI** | |
| --- | --- | --- | --- |
| cZNF292 | 0.789 | 0.702 | 0.861 |
| cSRSF4 | 0.584 | 0.488 | 0.681 |
| hypertensive | 0.56 | 0.473 | 0.636 |
| smoke | 0.648 | 0.564 | 0.721 |
| gender | 0.579 | 0.506 | 0.659 |
| age | 0.534 | 0.436 | 0.633 |
| BMI | 0.598 | 0.493 | 0.7 |
| heart-rate | 0.529 | 0.432 | 0.628 |
| systolic blood pressure | 0.505 | 0.412 | 0.616 |
| diastolic blood pressure | 0.521 | 0.419 | 0.612 |
| CM+cZNF292 | 0.832 | 0.754 | 0.888 |
| CM+cSRSF4 | 0.732 | 0.641 | 0.807 |
| cZNF292+hypertensive | 0.789 | 0.702 | 0.858 |
| cZNF292+smoke | 0.795 | 0.71 | 0.863 |
| cZNF292+gender | 0.793 | 0.706 | 0.859 |
| cZNF292+age | 0.793 | 0.699 | 0.858 |
| cZNF292+BMI | 0.814 | 0.734 | 0.877 |
| cZNF292+heart-rate | 0.794 | 0.707 | 0.864 |
| cZNF292+SBP | 0.789 | 0.698 | 0.856 |
| cZNF292+DBP | 0.789 | 0.695 | 0.854 |

**Table S4. ROC results for predicting UA and AMI.**

| **Predictors** | **AUC** | **95%CI** | |
| --- | --- | --- | --- |
| cZNF292 | 0.746 | 0.612 | 0.844 |
| cSRSF4 | 0.678 | 0.497 | 0.812 |
| gender | 0.501 | 0.417 | 0.639 |
| age | 0.632 | 0.435 | 0.799 |
| heart-rate | 0.535 | 0.403 | 0.699 |
| systolic blood pressure | 0.546 | 0.405 | 0.685 |
| diastolic blood pressure | 0.642 | 0.484 | 0.776 |
| BMI | 0.59 | 0.355 | 0.804 |
| smoke | 0.623 | 0.456 | 0.759 |
| CM+cSRSF4 | 0.827 | 0.705 | 0.91 |
| cZNF292+gender | 0.756 | 0.565 | 0.872 |
| cZNF292+age | 0.748 | 0.607 | 0.846 |
| cZNF292+heart-rate | 0.788 | 0.678 | 0.881 |
| cZNF292+SBP | 0.749 | 0.618 | 0.848 |
| cZNF292+DBP | 0.757 | 0.628 | 0.863 |
| cZNF292+BMI | 0.785 | 0.654 | 0.883 |
| cZNF292+smoke | 0.747 | 0.625 | 0.847 |

**Table S5. ROC results for predicting ACS and non-ACS.**

| **Predictors** | **AUC** | **95%CI** | |
| --- | --- | --- | --- |
| cZNF292 | 0.781 | 0.694 | 0.851 |
| cSRSF4 | 0.476 | 0.378 | 0.57 |
| hypertensive | 0.572 | 0.489 | 0.669 |
| diabetes | 0.537 | 0.459 | 0.611 |
| smoke | 0.641 | 0.55 | 0.722 |
| gender | 0.603 | 0.524 | 0.683 |
| age | 0.512 | 0.409 | 0.621 |
| BMI | 0.654 | 0.549 | 0.752 |
| heart-rate | 0.558 | 0.466 | 0.659 |
| systolic blood pressure | 0.529 | 0.421 | 0.626 |
| diastolic blood pressure | 0.542 | 0.444 | 0.633 |
| CM+cSRSF4 | 0.843 | 0.76 | 0.9 |
| cZNF292+hypertensive | 0.769 | 0.67 | 0.843 |
| cZNF292+diabetes | 0.785 | 0.698 | 0.849 |
| cZNF292+smoke | 0.781 | 0.698 | 0.851 |
| cZNF292+gender | 0.786 | 0.705 | 0.855 |
| cZNF292+age | 0.794 | 0.705 | 0.861 |
| cZNF292+BMI | 0.78 | 0.686 | 0.852 |
| cZNF292+heart-rate | 0.811 | 0.726 | 0.876 |
| cZNF292+SBP | 0.787 | 0.699 | 0.855 |
| cZNF292+DBP | 0.781 | 0.684 | 0.853 |

**Table S6. The characteristics of all patients with different levels of cZNF292.**

| **Variable** | **Low level** | **High level** | ***P value*** |
| --- | --- | --- | --- |
| N | 88 | 50 |  |
| **Demographic characteristics** |  |  |  |
| Male sex (% male) | 61(69.3) | 45(90.0) |  |
| Age (yrs) | 62.18±12.27 | 65.42±11.10 | 0.126 |
| BMI (kg/m2) | 20.00-23.98(21.55) | 19.00-22.50(20.60) | 0.065 |
| Heart rate (bpm) | 70.00-87.50(80.00) | 74.75-87.75(80.00) | 0.537 |
| SBP (mmHg) | 129.01±18.30 | 131.76±23.47 | 0.477 |
| DBP (mmHg) | 73.00-83.50(80.00) | 69.75-86.25(80.00) | 0.761 |
| **Biochemical examinations** |  |  |  |
| Hemoglobin (g/dL) | 128.00-151.00(141.00) | 130.00-155.25(145.00) | 0.527 |
| RBC(*10^12/L) | 4.57±0.52 | 4.62±0.51 | 0.640 |
| WBC(*10^9/L) | 7.39±2.59 | 8.78±2.64 | 0.003 |
| BPC(*10^9/L) | 205.13±61.40 | 205.02±54.23 | 0.992 |
| Neutrophil (%) | 64.72±11.72 | 69.41±12.69 | 0.030 |
| Potassium (mM) | 3.74±0.38 | 3.79±0.41 | 0.506 |
| Sodium (mM) | 141.72±2.47 | 140.46±3.15 | 0.017 |
| NT-proBNP (pg/mL) | 56.40-843.10(270.30) | 499.60-1832.75(1150.00) | 0.013 |
| hs-cTnI (ng/ml) | 0.010-11.588(0.034) | 5.730-78.000(42.098) | <0.001 |
| Myoglobin (ng/ml) | 17.90-98.60(30.20) | 73.20-627.40(181.45) | <0.001 |
| CK-MB (ng/ml) | 1.20-46.13(2.20) | 12.15-293.78(140.60) | <0.001 |
| D-dimer (mg/L) | 0.19-0.45(0.27) | 0.19-0.59(0.30) | 0.293 |
| CRP (mg/dL) | 0.59-3.75(1.57) | 0.62-5.60(2.28) | 0.734 |
| TB(μmol/L) | 14.13±6.07 | 18.08±7.74 | 0.001 |
| DB(μmol/L) | 1.43-3.48(2.55) | 1.80-4.45(3.15) | 0.123 |
| TC(mmol/L) | 4.52±1.14 | 4.50±1.42 | 0.914 |
| TG(mmol/L) | 1.04-1.86(1.28) | 0.90-1.71(1.12) | 0.481 |
| HDL-C (mM) | 1.04±0.22 | 1.02±0.23 | 0.743 |
| LDL-C (mM) | 3.10±0.90 | 3.12±1.18 | 0.910 |
| TP(g/L) | 66.78±5.99 | 66.28±6.19 | 0.644 |
| ALB (g/L) | 39.49±3.84 | 38.86±4.02 | 0.365 |
| ALT (U/L) | 14.00-36.75(22.50) | 19.75-45.25(32.50) | 0.863 |
| AST (U/L) | 18.00-57.75(26.50) | 23.00-184.75(66.00) | 0.003 |
| CK (ng/ml) | 64.25-385.00(100.50) | 79.75-1333.25(478.00) | 0.004 |
| BUN (mM) | 4.33-6.31(5.20) | 4.53-7.29(5.61) | 0.403 |
| Creatinine (µM) | 66.25-85.75(75.50) | 75.00-98.25(84.50) | 0.003 |
| Uric acid (µM) | 375.05±102.63 | 388.84±139.11 | 0.507 |

All data were presented as an absolute number (percentage), the mean (standard deviation) or the 25th-75th percentile (median).SBP, systolic blood pressure; DBP, diastolic blood pressure; RBC, red blood cell; WBC, white blood cell; BPC, blood platelet cell; CRP, C-Reactive Protein; TB, total bilirubin; DB, direct bilirubin; TC, total cholesterol; TG, triglyceride; HDL-C, high-density lipoprotein; LDL-C, lowdensity lipoprotein; TP, total protein; ALB, albumin; ALT, alanine amino transferase; AST, aspartate amino transferase; CK, creatine kinase; BUN, blood urea nitrogen.

**Table S7. Correlation analysis between blood cZNF292 and clinical experimental variables.**

| **Variable** | **R** | ***P value*** |
| --- | --- | --- |
| Age (yrs) | 0.034 | 0.691 |
| BMI (kg/m2) | -0.179 | 0.037 |
| Heart rate (bpm) | 0.048 | 0.577 |
| SBP (mmHg) | 0.080 | 0.352 |
| DBP (mmHg) | -0.024 | 0.783 |
| Hemoglobin (g/dL) | 0.206 | 0.015 |
| RBC(*10^12/L) | 0.181 | 0.034 |
| WBC(*10^9/L) | 0.311 | <0.001 |
| BPC(*10^9/L) | 0.061 | 0.476 |
| Neutrophil (%) | 0.212 | 0.013 |
| Potassium (mM) | 0.045 | 0.602 |
| Sodium (mM) | -0.232 | 0.006 |
| NT-proBNP (pg/mL) | 0.349 | <0.001 |
| hs-cTnI (ng/ml) | 0.494 | <0.001 |
| Myoglobin (ng/ml) | 0.445 | <0.001 |
| CK-MB (ng/ml) | 0.457 | <0.001 |
| D-dimer (mg/L) | 0.044 | 0.615 |
| CRP (mg/dL) | 0.024 | 0.776 |
| TB(μmol/L) | 0.339 | <0.001 |
| DB(μmol/L) | 0.173 | 0.042 |
| TC(mmol/L) | 0.038 | 0.661 |
| TG(mmol/L) | -0.139 | 0.104 |
| HDL-C (mM) | -0.033 | 0.700 |
| LDL-C (mM) | 0.067 | 0.435 |
| TP(g/L) | -0.017 | 0.843 |
| ALB (g/L) | -0.045 | 0.598 |
| ALT (U/L) | 0.197 | 0.020 |
| AST (U/L) | 0.286 | 0.001 |
| CK (ng/ml) | 0.250 | 0.003 |
| BUN (mM) | 0.086 | 0.317 |
| Creatinine (µM) | 0.227 | 0.007 |
| Uric acid (µM) | 0.027 | 0.756 |

SBP, systolic blood pressure; DBP, diastolic blood pressure; RBC, red blood cell; WBC, white blood cell; BPC, blood platelet cell; CRP, C-Reactive Protein; TB, total bilirubin; DB, direct bilirubin; TC, total cholesterol; TG, triglyceride; HDL-C, high-density lipoprotein; LDL-C, lowdensity lipoprotein; TP, total protein; ALB, albumin; ALT, alanine amino transferase; AST, aspartate amino transferase; CK, creatine kinase; BUN, blood urea nitrogen.

**Table S8. The sequences of primers used for RT-PCR and semiquantitative PCR.**

| **Gene** | **Primer sequence (5’**-**3’)** |
| --- | --- |
| cZNF292 Forward | GCTCAAGAGACTGGGGTGTG |
| cZNF292 Reverse | AGTGTGTGTTCTGGGGCAAG |
| cAFF1 Forward | GCCAAGCTCACCAAACTGAA |
| cAFF1 Reverse | CCTGGTTGCGTCTTTCCTTC |
| cDENND4C Forward | CTTCATGACCCACCACAAGATG |
| cDENND4C Reverse | GGGTGTGGCTAGGATCACTTC |
| cTHSD1 Forward | TCTGGACGTGAGAAAGGACA |
| cTHSD1 Reverse | TCAGTGTCCCATTAGCACCA |
| cSRSF4 Forward | AAGACAAGCCAGGTTCCAGA |
| cSRSF4 Reverse | TTTTGCGTCCCTTGTGAGC |
| ZNF292 Forward | GCAAAGCTGTGTTCTGACCA |
| ZNF292 Reverse | CTTGTTGGAGCTGACGTGAC |
